# Editorial: The first 1000 days: window of opportunity for child health and development

**DOI:** 10.3389/fnut.2025.1673003

**Published:** 2025-08-18

**Authors:** Shoba Suri, Giovanna Verlato, Subhasree Ray

**Affiliations:** ^1^Observer Research Foundation, New Delhi, India; ^2^Pediatric Nutrition Service-Neonatal Intensive Care Unit, University Hospital of Padova, Padova, Italy; ^3^TVS Motor Company (India), Chennai, India

**Keywords:** 1,000 days of life, child health, child development, infant and young child feeding, social determinants, environmental factors, healthcare

## Introduction

The first 1,000 days of a child's life, spanning from conception to their second birthday, represent a pivotal period that shapes their future health, cognitive abilities, and overall wellbeing. This critical window is a time of extraordinary growth and development, particularly for the brain, which undergoes rapid formation of neural connections that lay the foundation for lifelong learning and health (1). During this phase, a child's body grows at a remarkable pace, and their immune system begins to mature, making proper nutrition, healthcare, and socio-emotional support indispensable. Research underscores that interventions during this period can have profound, long-lasting effects, while neglect or adverse conditions can lead to irreversible consequences, such as stunted growth, cognitive deficits, and compromised immunity (2, 3). In this Research Topic, we invited researchers, public health practitioners, and policy experts to share their work related to nutrition, caregiving, social protection programs, and early child development within the first 1,000 days. The published articles offer a comprehensive view of the multifactorial influences and interventions that shape early childhood outcomes.

## The importance of the first 1,000 days

The concept of the first 1,000 days as a “window of opportunity” is grounded in extensive scientific evidence demonstrating that early interventions can yield significant dividends for individuals and societies. Proper nutrition during this period is paramount. Breastfeeding, for instance, provides essential nutrients and antibodies that bolster a child's immune system and cognitive development (4). A diverse, nutrient-rich diet further supports physical growth and prevents deficiencies that could impair development (5). Access to clean water, sanitation, and healthcare services—such as vaccinations and regular check-ups—ensures that children are protected from preventable diseases and developmental setbacks. Beyond physical health, the socio-emotional environment plays a critical role. Responsive caregiving, a safe living environment, and opportunities for stimulation foster emotional security and cognitive growth, setting the stage for resilience and adaptability in later life.

The consequences of failing to provide adequate support during this period are severe. Malnutrition, for example, can lead to stunting, which affects not only physical growth but also cognitive capacity and economic productivity in adulthood (6). Similarly, lack of healthcare access can result in untreated illnesses that hinder development, while exposure to environmental toxins or inadequate caregiving can impair brain development and emotional wellbeing. Investing in the first 1,000 days is thus not only a moral imperative but also an economic one, as healthier children grow into more productive adults, contributing to stronger communities and equitable societies.

## Challenges to optimizing child health and development

Despite the clear importance of the first 1,000 days, numerous challenges hinder efforts to ensure that all children receive the necessary support. Malnutrition remains a pervasive issue, particularly in low- and middle-income countries, where millions of children lack access to adequate nutrition (7). This leads to conditions such as wasting, stunting, and underweight, which have long-term consequences for health and development. Limited access to healthcare services, especially in remote or low-income areas, exacerbates these issues, as families struggle to obtain vaccinations, prenatal care, or treatment for illnesses. Poverty compounds these challenges, restricting access to nutritious food, clean water, and safe living environments. Additionally, a lack of education and awareness among parents and caregivers about the importance of the first 1,000 days can result in missed opportunities for intervention (8). Environmental factors, such as exposure to pollutants or inadequate sanitation, further threaten child health, particularly in resource-constrained settings.

Addressing these challenges requires coordinated, evidence-based policies and programs that prioritize early childhood development. Governments, NGOs, and communities must work together to improve access to nutrition, healthcare, and education while addressing systemic issues like poverty and environmental degradation.

## Insights from the Research Topic

The Research Topic, comprising 19 chapters, offers a multifaceted examination of the first 1,000 days, drawing on empirical research from diverse global contexts. Each chapter contributes to a deeper understanding of the factors influencing child health and development and proposes strategies to address the associated challenges. Below, we highlight key themes and findings from select chapters, illustrating the breadth and depth of this critical research.

## Nutrition and feeding practices

Nutrition is a cornerstone of child development during the first 1,000 days, and several chapters in the topic address this Research Topic. For instance, S. Calgaro et al. evaluates feeding practices in the first 12 months of life in Beira, Mozambique, highlighting the link between suboptimal feeding practices (inadequate breastfeeding during complementary feeding) and poor nutritional outcomes in low-resource urban settings (Calgaro et al.). Similarly, Hailemariam Mamo Hassen examines trends in exclusive and predominant breastfeeding practices in Ethiopia over two decades, underscoring the importance of sustained efforts to promote breastfeeding as a cost-effective intervention for child health (Hassen). Rohini Saran et al. explores the role of fortification and breastfeeding in improving nutrition outcomes, emphasizing the need for scalable interventions to combat malnutrition (Saran et al.).

Maternal nutrition also plays a critical role, as evidenced by Teshale Fikadu et al., which investigates the nexus between maternal nutritional status and food consumption patterns in pregnant women in South Ethiopia (Fikadu T. et al.). The study reveals how maternal diets influence fetal development, highlighting the need for targeted nutritional support for pregnant women. Additionally, Li et al. examines how dietary preferences, such as a preference for mala-flavored foods in Chongqing, China, can lead to excessive gestational weight gain, mediated by high-carbohydrate diets, which may affect both maternal and child health (Li et al.).

## Healthcare and supplementation

Access to healthcare and appropriate supplementation is another critical focus of the Research Topic. Lian et al. investigates the effects of folic acid supplementation before conception on the immune function and anti-HBs levels of offspring born to HBsAg-positive mothers, demonstrating the protective role of preconception interventions (Lian et al.). Similarly, Yin et al. explores the necessity of gestational vitamin D supplementation, noting its dependence on ambient temperature and its implications for infant vitamin D status (Yin et al.). Baker et al. advocates for omega-3 long-chain polyunsaturated fatty acid (LC-PUFA) consumption during pregnancy to reduce the risk of preterm birth, emphasizing sustainable implementation strategies (Baker et al.). These findings highlight the importance of tailored healthcare interventions to address specific nutritional deficiencies and health risks during pregnancy and early childhood.

## Socio-emotional and environmental factors

The socio-emotional environment and caregiving practices are equally vital for child development. Alabdullah et al. uses a qualitative approach to explore factors influencing breastfeeding knowledge and practices among Saudi mothers, revealing the role of cultural barriers and the importance of education (Alabdullah et al.). Randhawa et al. examines the knowledge, attitudes, and practices of mothers and frontline health workers in Assam, India, emphasizing the need for community-based education to improve infant and young child feeding (Randhawa et al.). Benjamin et al. focuses on transforming hospital organizational culture to promote parent-child relationships, demonstrating how institutional changes can foster supportive environments for early childhood development (Benjamin et al.).

Environmental factors also receive attention. Song et al. investigates the relationship between maternal vitamin D status during pregnancy and infant gut microbiota, highlighting the interplay between maternal health and environmental influences on child development (Song et al.). Alamneh et al. explores recovery times from severe acute malnutrition among cholera-exposed and unexposed children in Ethiopia, underscoring the impact of environmental health crises on nutritional outcomes (Alamneh et al.).

## Policy and systemic interventions

Several studies address the role of policy and systemic interventions in improving outcomes during the first 1,000 days. Jagannath and Chakravarthy evaluates the impact of India's Pradhan Mantri Matru Vandana Yojna scheme, which improves access to maternal and child health services, leading to better health and nutritional outcomes (Jagannath and Chakravarthy). Fikadu et al. examines intra-household decision-making on child feeding in rural South Ethiopia, highlighting the social determinants that influence nutritional practices. These studies emphasize the need for policies that address both individual and systemic barriers to child health (Fikadu K. et al.).

## Addressing malnutrition and health disparities

The Research Topic also tackles the persistent challenge of malnutrition and health disparities. Tamir et al. uses spatial and multilevel analyses of the 2023 Senegal Demographic and Health Survey to map the prevalence and determinants of wasting among children under five, providing critical data for targeted interventions (Tamir et al.). Dassie et al. conducts a systematic review of factors influencing concurrent wasting, stunting, and underweight in low- and middle-income countries, offering insights into the complex interplay of nutritional deficiencies (Dassie et al.). De Rose et al. provides a practical overview of improving growth in preterm infants through nutrition, addressing a vulnerable population with unique needs (De Rose et al.).

## Moving forward: a call to action

The first 1,000 days represent a unique opportunity to shape the trajectory of a child's life, but realizing this potential requires overcoming significant challenges. The Research Topic provides a roadmap for action, highlighting the need for integrated approaches that combine nutrition, healthcare, education, and environmental interventions. Policymakers must prioritize investments in maternal and child health programs, ensuring access to nutritious food, clean water, and healthcare services, particularly in low-resource settings. Community-based education campaigns can empower parents and caregivers with the knowledge and tools to support their children's development. Additionally, addressing systemic issues like poverty and environmental degradation is essential to creating equitable opportunities for all children.

The research presented under the Research Topic underscores the global nature of these challenges and the need for context-specific solutions. From urban Mozambique to rural Ethiopia, from Saudi Arabia to China, the studies highlight the diverse factors influencing child health and development. By synthesizing these insights, the Research Topic calls for a collaborative effort among governments, NGOs, healthcare providers, and communities to ensure that every child has the opportunity to thrive during the first 1,000 days.

In conclusion, the first 1,000 days are a window of opportunity that we cannot afford to miss. By investing in nutrition, healthcare, and supportive environments during this period, we can lay the foundation for healthier, more productive, and equitable societies. The Research Topic serves as both a call to action and a blueprint for change, urging stakeholders to prioritize the health and wellbeing of children in their earliest years. Together, we can transform the promise of the first 1,000 days into a reality for all children, ensuring a brighter future for generations to come.
